# Cytotoxic and Antimigration Activity of *Etlingera alba* (A.D.) Poulsen Rhizome

**DOI:** 10.1155/2021/6597402

**Published:** 2021-12-28

**Authors:** W. Wahyuni, Ajeng Diantini, Mohammad Ghozali, Anas Subarnas, Euis Julaeha, Riezki Amalia, I. Sahidin

**Affiliations:** ^1^Department of Pharmacology and Clinical Pharmacy, Faculty of Pharmacy, Universitas Padjadjaran, Jatinangor, Indonesia; ^2^Faculty of Pharmacy, Universitas Halu Oleo, Kendari, Indonesia; ^3^Department of Biomedical Sciences, Faculty of Medicine, Universitas Padjadjaran, Jatinangor, Indonesia; ^4^Department of Chemistry, Faculty of Mathematics and Natural Sciences, Universitas Padjadjaran, Jatinangor, Indonesia

## Abstract

*Etlingera alba* is one of the *Etlingera* plants that might have anticancer activity. This study aims to investigate the cytotoxic and antimetastatic activity of *E. alba* rhizome fractions and migration cell assay against MDA-MB-231 cell lines, which are used for triple-negative breast cancer (TNBC) treatment assay. The cytotoxic activity was assayed using CCK-8 assay, while the antimetastatic was assayed using migration cell assay for the fractions A–F. They were followed by LCMS/MS profiling to determine the chemical contents in the most active fraction. According to results obtained, fraction B was the most active fraction for cytotoxic activity with an IC_50_ value of 65.43 *μ*g/mL, while fraction *E* was the most active fraction for antimetastasis activity against migration rate doses of 50, 100, and 200 ppm which were 6.80, 3.66, and 3.00%, respectively. Several compounds in fraction B, such as rengyolone, licochalcone A, sugiol, and spinasterol, might have been known to have activity against cancer cells, as well as aschantin and lirioresinol B dimethyl ether from fraction E. In conclusion, the chemical components from *E. alba* rhizome fractions provided potency for discovering new agents for cancer treatment, specifically for TNBC.

## 1. Introduction

Cancer is the first or second leading cause of death, mainly worldwide, according to WHO (World Health Organization). The incidence and mortality gradually increase each year, including breast cancer. In 2020, the new cases and deaths reported were 2.261.419 cases (11.7%) and 684.999 deaths (6.9%) because of breast cancer [1]. Breast cancer is commonly metastatic cancer, thus it can transfer to other organs and cause death [2].

Triple-negative breast cancer (TNBC) is a kind of breast cancer diagnosed in 15–20% of breast cancer cases. It is categorized with lack or absence of the presence of estrogen (ER), progesterone (PR), and HER-2 (human epidermal growth receptor-2) receptor expression. The TNBC contributes to 170,000 cases with more aggressive behavior, high risk of recurrence, and high mortality than the non-TNBC [3–6].

In treating breast cancer, surgery, radiation therapy, and chemotherapy are the options. The chemotherapy is administered to shrink the tumor before the surgery. However, they can cause many side effects, such as nausea and vomiting, neutropenia, rash and redness, nephrotoxicity, and cardiotoxicity[7]. Therefore, discovering a new agent with the lowest side effect and high efficacy is required because the TNBC has poor prognosis than other breast cancer types. Many compounds from terrestrial plants are the potential for providing anticancer activity. For example, chemotherapy agents such as taxol, vincristine, and vinblastine are from terrestrial plants [6, 8, 9].

*Etlingera alba* (A.D.) Poulsen is one of the newest Etlingera species found in Southeast Sulawesi, and not many studies are conducted on it. The *E. alba* is well known medicinal herb similar to *E. elatior*, mainly used to treat wounds, as deodorant, and as earache medicine. A previous study showed that *E. alba* rhizome extract has antibacterial and anti-inflammatory properties [10, 11]. *E. alba* becomes a promising candidate as a source of therapeutical agents. Many studies showed that species that belong to similar genus provide similar activities. For example, *E. elatior* is a species that belongs to a similar genus with *E. alba.* It provides antibacterial, antioxidant, anti-inflammatory, and antipyretic properties. Secondary metabolites, including phenolics, alkaloids, flavonoids, steroids, saponin, and volatile oil, are responsible for providing these properties [11].

*E. elatior* flower provides cytotoxic activity against MDA-MB-231 cell line with IC_50_ 196.2 *μ*g/mL [12], *E. belalongensis* rhizome and stem with IC_50_ 51.00 ± 4.24 and 74.00 ± 2.83 *μ*g/mL, respectively [13], and *E. velutina* rhizome and stem with IC_50_ 67.00 ± 9.89 and 89.50 ± 14.85 *μ*g/mL, respectively [13]. In addition, bioactive compound trans-4-methoxycinnamaldehyde (4-MCA) identified in *E. pavieana* rhizome exhibited IC50 values 39.33±1.53 *μ*g/mL against MDA-MB-231 cell lines [14]. MDA-MB-231 cell is an invasive ductal/breast carcinoma cell which is one of the tools for researching TNBC because it lacks amplifying estrogen receptor (ER) and progesterone receptor (PR) expression, as well as HER2 (human epidermal growth factor receptor 2). It is highly metastatic and aggressive [15, 16]. Thus, we aim to investigate the cytotoxic activity of fractions from extract *E. alba* rhizomes by using CCK-8 assay against MDA-MB-231 cell lines and their metastatic activity by using migration cell assay.

## 2. Materials and Methods

### 2.1. Plant Material

*Etlingera alba* rhizomes were obtained from Punggaluku of South Konawe Regency in Southeast Sulawesi (4019′26″S 122028′58″E, 325 m). The rhizomes were dried at 40°C and direct sunlight was avoided. The rhizomes were powdered and stored in a sealed jar for further analysis.

#### 2.1.1. Cell Lines Preparation and Culture

MDA-MB-231 cell line was grown in 100 mm plate in CO_2_ incubator and observed under an inverted microscope. The cell line was seeded in 6-well plates with 2 mL of media per well. A total of 2 × 10^4^ cells/well were needed for cytotoxicity assay and migration assay using scratch wound-healing assay. Cell distribution was observed under an inverted microscope to obtain the even distribution until attaining ∼70–80% of confluence.

### 2.2. Chemical Reagents

Ethanol 95% (technical grade), silica gel 60 (0.063–0.200 mm) Merck 107734, silica gel 60 GF254 Merck 1.07730, TLC silica gel 60 F_254_ Merck 1.05554.0001, n-hexan (technical grade), ethyl acetate (technical grade), methanol (technical grade), formic acid, water, acetonitrile, Dulbecco's modified Eagle medium (DMEM)-high glucose (Sigma); fetal bovine serum (Sigma); trypsin TrypLE (Gibco); penicillin-streptomycin (Sigma); phosphate buffer saline 10X (Lonza); cell counting kit-8 (product code: CK04).

### 2.3. Apparatus

Vacuum rotary evaporator (Buchi®), vacuum liquid chromatography (VLC), LCMS/MS, Xevo G2-XS QTOF (Waters Corporation, Milford, USA), microplate reader, 96-well microplate; 6-well plate (Nest); plate 100 mm (Nest); serological pipettes 5 mL, 10 mL, and 25 mL (Nest); micropipette (Eppendorf) in various measurements, pipette tips in 1000 *μ*L (GenFollower), 200 *μ*L (GenFollower), and 10 *μ*L (Biologix); CO_2_ incubator (Thermo Scientific); centrifuge tubes 15 mL and 50 mL (Nest); haemocytometer (Assistant-Germany); light microscope Axio Vert.A.1 (Zeiss) camera build-in, and Zen microscope software (Zeiss); biological safety cabinet-level II (ESCO); centrifuge PLC series; Multi-Vortex V-32 (Biosan).

### 2.4. Extraction and Fractionation

A total of 5.5 kg of powdered *E. alba* rhizome was macerated with 96% ethanol for 3 × 24 hrs at room temperature. The filtrate obtained was concentrated under a low-pressure vacuum with a rotary evaporator at 40°C in 160 g of concentrated extract (2.9%). The extract was impregnated into silica gel and fractionated using vacuum liquid chromatography (VLC, 10 × 5 cm) and the mixture of n-hexane: ethyl acetate with increased polarity (9 : 1 to 0 : 10 v/v) and pure MeOH till obtained 6 fractions. The fractions were fraction A (3,239 g); fraction B (4,345 g); fraction C (5,362 g); fraction *D* (1,563 mg); fraction *E* (4,432 mg); and fraction *F* (74,224 mg).

### 2.5. Cytotoxic Assay

The triple-negative breast cancer cell line (MDA-MB-231) was obtained from the collection of the Department of Pharmacology and Clinical Pharmacy, Universitas Padjadjaran. The cells were grown in Dulbecco's modified Eagle medium (Sigma-Aldrich, St. Louis, MO) containing 10% fetal bovine serum (Sigma-Aldrich, St. Louis, MO) and 1% penicillin-streptomycin (Sigma-Aldrich, St. Louis, MO). The cell line was seeded in a 96-well plate and cultured for 24 h at 37°C hrs in a humidified atmosphere of 5% CO_2_. The cells were refreshed with new media and treated with various concentrations of samples, cisplatin was used as control, and DMSO was used as blank. After 24 h, cell counting kit-8 (CCK) reagent (Dojindo, Rockville, MD, USA) was added, and the mixtures were incubated for 4 h. The absorbances were measured using a Tecan Infinite spectrophotometer (*λ* 450 nm).

### 2.6. Migration Cell Assay

The migration rates of MDA-MB-231 cells were assessed by the scratch assay method. The cells were seeded in 6-well plates, and after 70–80% confluency, the media was replaced with a low FBS growth medium (concentration 0.5%) and cultured at 37°C in a humidified atmosphere of 5% CO_2_. After 24 h, the monolayer cells were scratched with sterile yellow micropipette tips, and the cells' debris was washed with PBS. The cells were treated with various concentrations of a sample by diluting with serum-free DMEM. The cells treated with cisplatin were used as a positive control. The scratch induced that represented wound was photographed at 0 h using phase-contrast microscopy at ×40 magnification at 0 h. After 24 h of incubation, the second set of images was photographed, and the percentage of the closed area was measured and compared with the value obtained at 0 h. An increase in the percentage of the closed area indicated the migration of cells. Experiments were performed in a triplicate manner, and the data were recorded and analyzed statistically using SPSS.

To determine the migration rate, the migration percentage (%) was calculated as

(1) $$\begin{array}{c} \%\text{migration}=\frac{\left(\text{diameter at }0-\text{hrs}\right)-\left(\text{diameter at }24-\text{hrs}\right)}{\left.\left(\text{diameter at }0-\text{hrs}\right)\right]}\times 100\%. \end{array}$$

### 2.7. LCMS/MS

Fraction B and *E* were identified chemically using LCMS/MS analysis, Xevo G2-XS QTOF (Waters Corporation, Milford, USA), equipped with an electrospray ionization source (ESI). The HSS T3 C18 column was reversed phase (2.1 × 100 mm, particle size 1.8 m) at 40°C. The mobile phase consisted of A (0.1% formic acid in water) and B (acetonitrile in 0.1% formic acid). Elution gradient was at a flow rate of 0.3 mL/min with an injection volume of 1 L. The gradients were 5% B (0–8 min), 40% B (8–11 min), and 100% B (11–16 min). Data range from 50–1200 m/z. The applied source temperature was 120°C, and the desolvation gas flow was 1000 L/hour with a desolvation temperature of 500°C. The capillary voltage was at 2.0 kV, and the cone voltage was 30 V. Low energy scan was at 6.00 eV and high energy scan at 40 eV. All LCMS data were processed, peaked, and analyzed. The variables of interest were then identified using the UNIFI software.

## 3. Results and Discussion

**Figure 1:**
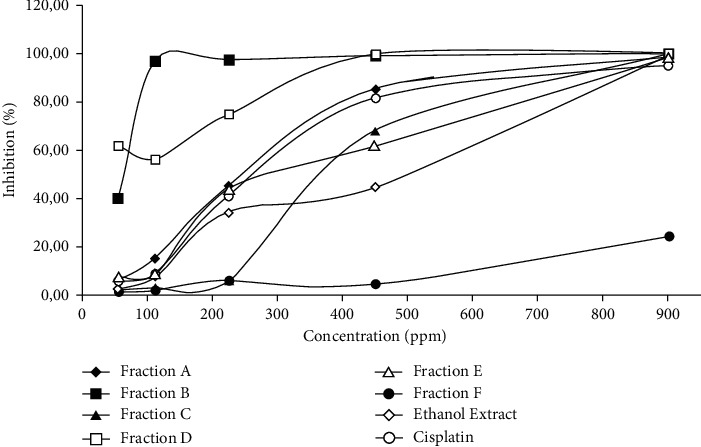
Inhibitory activity of extract and fractions on MDA-MB-231 cell line growth in various concentrations.

The inhibition of each fraction against MDA-MB-231 cells exhibited various activities. The chemical compounds in each fraction influence these results. Each chemical compound will have a molecular structure with specific physical and chemical properties. The different properties of each of these molecular structures, of course, provide different pharmacological effects. According to Figure 1, the extract and fractions exhibited concentration-dependent inhibition with the higher concentration which gave higher inhibition activity. Fraction B provides better inhibitory activity similar to cisplatin than another fraction against MDA-MB-231 cell line with an IC_50_ value of 65.43 *μ*g/mL closest to cisplatin with an IC_50_ value of 53.37 *μ*g/mL. The IC_50_ values of extract, fraction A, fraction C, fraction D, fraction E, and fraction F were 453.36; 252.25; 497.98; 262.5; 345.3; and 1840.85 *μ*g/mL.

**Figure 2:**
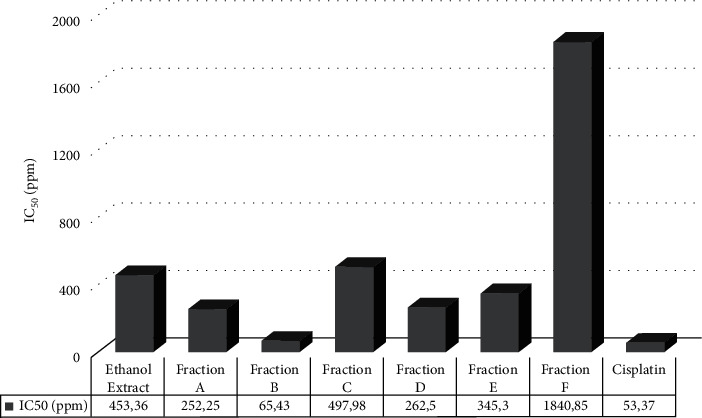
IC_50_ value of extract and fraction on MDA-MB-231 cell growth.

Analog to Figure 2, cisplatin is used as control due to its ability to disrupt the cell division and trigger cell death by forming cross-links with guanine bases of DNA double helix chain leading to transcription and DNA replication interfered. Like cisplatin, the chemical compounds contained in fraction B certainly have an essential role in cytotoxic effects on MDA-MB-231 cells.

**Figure 3:**
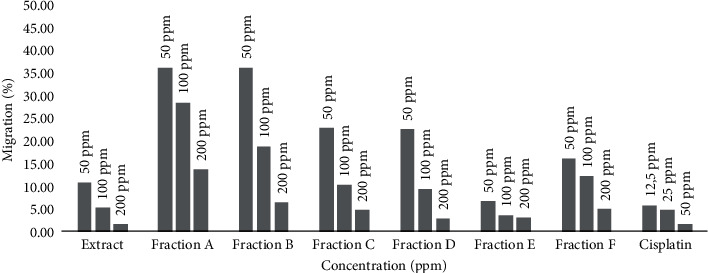
The migration rate in percentage for MDA-MB-231 cell line.

**Figure 4:**
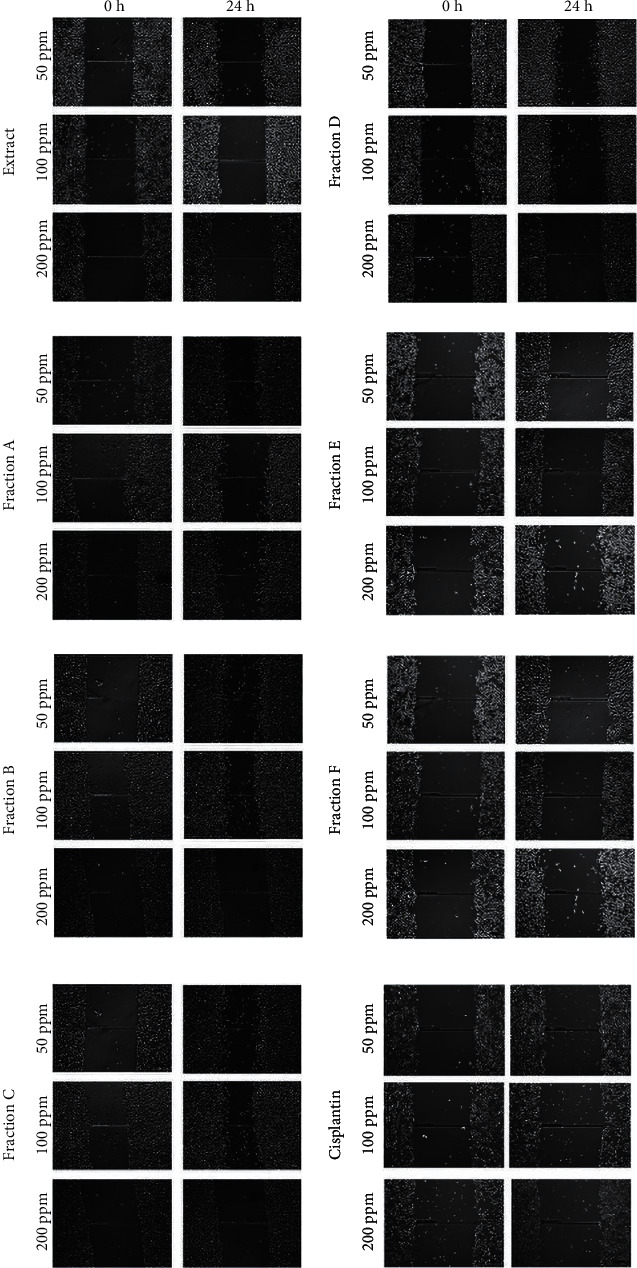
*In vitro* scratch assay (×40 magnification) of MDA-MB-231 cells towards extract, fractions, and cisplatin in various concentrations (50 ppm; 100 ppm; and 200 ppm).

Migration is a critical event in cancer progression, specifically in metastasis; thus, the extract of *E. alba* rhizome and its fractions were evaluated [17]. The cell migration rate is shown in Figure 3, in which cells migrate towards the provisional induced in 24 hours [18].  Figure 4 shows that cells migrate in 24 hrs. The study results demonstrated that fraction E showed the lowest migration rate compared to other fractions and the extract itself by the concentrations 50, 100, and 200 ppm in a dose-dependent manner, which were 6.80, 3.66, and 3.00%. They were not significant to cisplatin as control, which was 5.66, 4.84, and 1.61%, respectively. It suggests that fraction E can prevent the metastasis that produces secondary tumors in an organism [19].

A previous study showed that cisplatin could prevent metastasis by blocking the early step of EMT (epithelial-mesenchymal transition). Cisplatin blocks the cell migration induced by transforming growth factor-beta (TGF-*β*), which acts as a tumor promoter that leads to metastasis. On MDA-MB-231 cells, cisplatin blocks the filopodia formation associated with dynamic cytoskeleton rearrangement induced by TGF-*β* cells during cell movement [20]. The fraction F provides similar activity to control, which is cisplatin observed 12 hr after treatment. The mechanism itself is similar to cisplatin by blocking the filopodia formation in cell movement.

**Figure 5:**
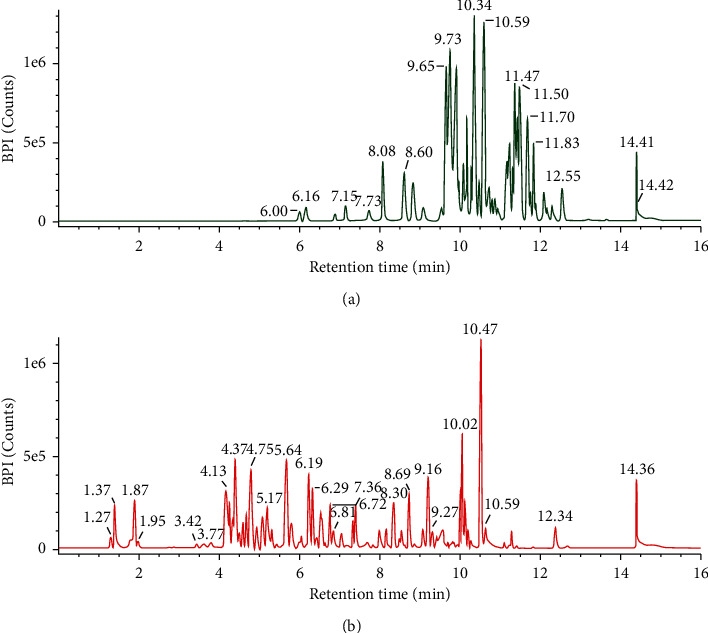
LCMS chromatogram of the chemical constituents of fraction B (a) and fraction E (b).

**Figure 6:**
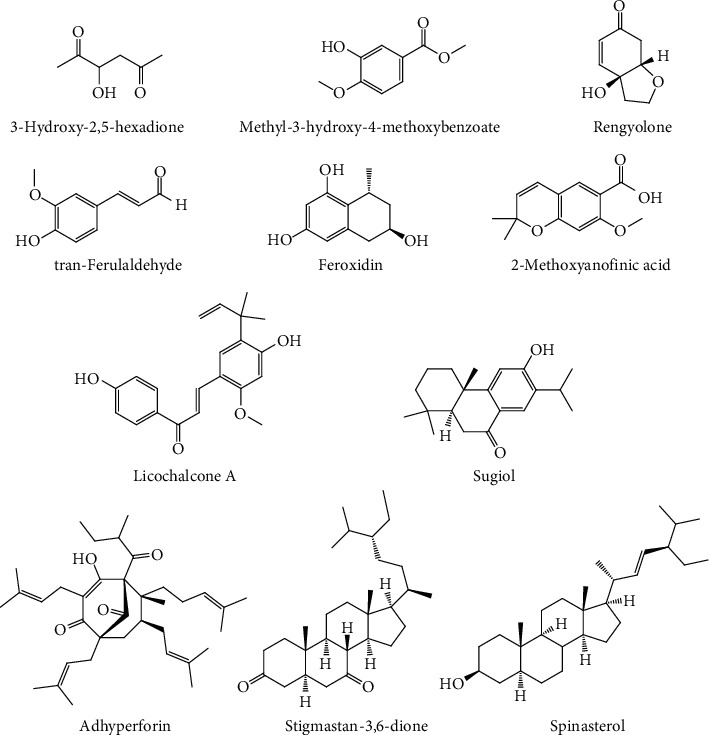
Structure molecule of the chemical constituents of fraction B.

**Figure 7:**
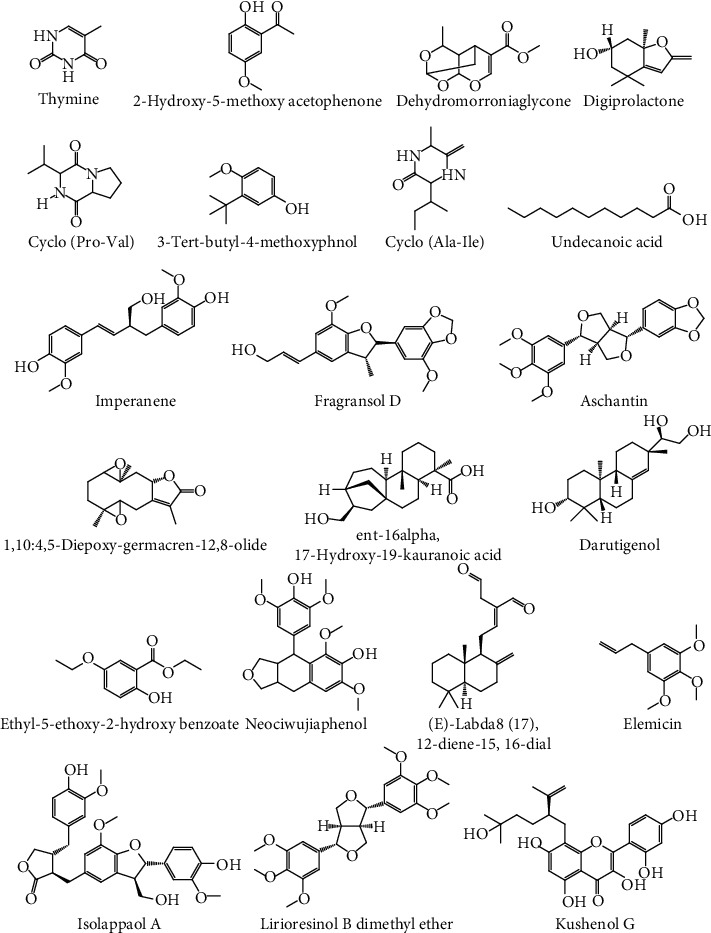
Structure molecule of the chemical constituents of fraction E.

Analysis of the chemical content in fractions B and E using LCMS/MS showed separated compounds based on their polarity. The initial compounds that appear first are polar and are increasingly nonpolar as the retention time on the chromatogram increases [21]. The chromatogram at each retention time (Figures 5(a) and 5(b)) obtained was processed using UNIFI software so that peak height, area, and fragmentation spectra of chemical compounds could be known so that the formula and molecular structure of the compound could be understood. Each chromatogram peak induces one chemical compound. The results obtained in the LCMS/MS analysis are presented in Table 1, while the chemical compounds' structure is shown in Figures 6 and 7, respectively.

LCMS-MS profiling of fraction B and fraction E is shown in Figures 5(a) and 5(b), in which retention time (Rt) was observed from 5.99 to 11.69 min for fraction B and 1.38 to 9.28 min for fraction E. For fraction B (Table 1), compound 1 in positive mode ions appeared at time 5.99 min with peak [M+Na]^+^ at m/z 153.0534, with mass 130 which confirmed it was 3-hydroxy-2,5-hexadione with formula C_6_H_10_O_3_. This process can be applied to determine the other compounds seen in Tables 1 and 2, respectively, for fractions B and E. The structure of each compound is available in Figures 6 and 7, respectively.

Although each fraction contains such compounds, not all of them provide anticancer activity. Sometimes they even synergize with each other to provide this activity. Several compounds in fraction B, such as rengyolone [22], licochalcone A [23], sugiol [24, 25], and spinasterol [26], have been known to have activity against cancer cells. For fraction E, thymine [27], aschantin [28], lirioresinol B dimethyl ether [29], and others have potency as anticancer, as well.

For the compound licocalchone A, the antiproliferative and apoptotic effects of MDA-MB-231 cancer cells have been investigated. Licocalchone A provides an antiproliferative activity by inducing cell cycle arrest at G0/G1 in MDA-MB-231 cells [23, 30]. In addition, licocalchone A exhibits apoptotic effects in MDA-MB-231 cells by inducing cleaved caspase-3 and caspase-8 and promoting caspase-3 activity in MDA-MB-231 cells through partial extrinsic signal pathway activation [23, 30]. It also provides an antimetastatic effect, although our finding resulted in the antimetastatic effect by wound-healing methods which was not better than fraction E. Licochalcone A decreased phosphorylation of AKT, JNK, *p*38, and NF-*κ*B. It also enhances the expressions of E-cadherin and reduces vimentin levels in MDA-MB-231 cells. This activity was in a concentration-dependent manner [23]. Thus, licochalcone A is a chemical component in fraction B, influencing the cytotoxic effect on MDA-MB-231 cells.

Fraction E demonstrated the antimetastasis effect through antimigration activity compared to other fractions because of the lirioresinol B dimethyl ether (LBDE) contained in fraction E. LBDE provides antimetastasis and bone microenvironment by inhibiting parathyroid hormone-related protein (PTHrP), matrix metallopeptidase 9 (MMP-9), and cathepsin K activities in breast cancer cells and resorption of osteoclastic bone [29]. LBDE belongs to tertrahydrofurofuran lignans that prevent metastasis by suppressing TGF-*β* that induced cancer cell viability, migration, invasion, and PTHrP expression. PTHrP has a role in cancer progression and metastasis through a vicious cycle [29]. Fraction E also provides cytotoxic activity, although it was not efficacious than fraction B. This activity might be affected by NF-*κ*B signaling pathway inhibition [31].

## 4. Conclusions

Our finding suggests that fraction B of *E. alba* rhizome extract has an antiproliferative effect on the MDA-MB-231 cell lines. On the other hand, fraction E displayed better activity in a migration cell assay. Fraction B that suppresses the tumor growth with licocalchone A might provide this activity, but not as efficacious as cisplatin in blocking tumor growth. On the contrary, fraction E is still not more efficacious than cisplatin in suppressing tumor growth but has similar activity in blocking metastasis. The lirioresinol B dimethyl ether (LBDE) contained in this fraction E might provide this antimetastasis activity.

## Figures and Tables

**Table 1:** Chemical composition of fraction B.

No.	Observed Rt (min)	Observed (m/z) [(+)-ESI]	Neutral mass (Da)	Formula	Compound name
1	5.99	153.0534 [M+Na]^+^	130.06299	C_6_H_10_O_3_	3-Hydroxy-2,5-hexadione
2	6.16	183.0640 [M+H]^+^	182.05791	C_9_H_10_O_4_	Methyl-3-hydroxy-4-methoxybenzoate
3	7.15	155.0690 [M+H]^+^	154.06299	C_8_H_10_O_3_	Rengyolone
4	8.08	216.1736 [M+H]^+^	215.16740	C_15_H_21_N	Candidate mass C_15_H_21_N
5	8.61	179.0691 [M+H]^+^	178.06299	C_10_H_10_O_3_	tran-Ferulaldehyde
6	9.66	195.1004 [M+H]^+^	194.09429	C_11_H_14_O_3_	Feroxidin
7	9.74	235.0956 [M+H]^+^	234.08921	C_13_H_14_O_4_	2-Methoxyanofinic acid
8	9.82	339.1582 [M+H]^+^	338.15181	C_21_H_22_O_4_	Licochalcone A
9	10.12	233.1889 [M+H]^+^	232.18272	C_16_H_24_O	Candidate mass C_16_H_24_O
10	10.18	301.2154 [M+H]^+^	300.20893	C_20_H_28_O_2_	Sugiol
11	10.34	477.3655 [M+Na]^+^	454.37706	C_26_H_50_N_2_O_4_	Candidate mass C_26_H_50_N_2_O_4_
12	10.48	505.3973 [M+Na]^+^	482.40836	C_28_H_54_N_2_O_4_	Candidate mass C_28_H_54_N_2_O_4_
13	10.82	573.3923 [M+Na]^+^	550.40221	C_36_H_54_O_4_	Adhyperforin
14	11.32	429.3721 [M+H]^+^	428.36543	C_29_H_48_O_2_	Stigmastan-3,6-dione
15	11.43	413.3771 [M+H]^+^	412.37052	C_29_H_48_O	Spinasterol
16	11.50	639.4974 [M+Na]^+^	616.50668	C_39_H_68_O_5_	Candidate mass C_39_H_68_O_5_
17	11.69	615.4965 [M+H]^+^	614.49103	C_39_H_66_O_5_	Candidate mass C_39_H_66_O_5_

**Table 2:** Chemical composition of fraction E.

No.	Observed Rt (min)	Observed (m/z) [(+)-ESI]	Neutral mass (Da)	Formula	Compound name
1	1.38	113.0332 [M+H]^+^	112.02728	C_4_H_4_N_2_O_2_	Candidate mass C_4_H_4_N_2_O_2_
2	1.86	127.0489 [M+H]^+^	126.04293	C_5_H_6_N_2_O_2_	Thymine
3	4.16	237.1113 [M+H]^+^	236.10486	C_13_H_16_O_4_	Candidate mass C_13_H_16_O_4_
4	4.22	167.0692 [M+H]^+^	166.06299	C_9_H_10_O_3_	2-Hydroxy-5-methoxy acetophenone
5	4.37	227.0905 [M+H]^+^	226.08412	C_11_H_14_O_5_	Dehydromorroniaglycone
6	4.47	219.1005 [M+Na]^+^	196.10994	C_11_H_16_O_3_	Digiprolactone
7	4.56	197.1273 [M+H]^+^	196.12118	C_10_H_16_N_2_O_2_	Cyclo(Pro-Val)
8	4.75	203.1055 [M+Na]^+^	180.11503	C_11_H_16_O_2_	3-Tert-butyl-4-methoxyphenol
9	4.92	185.1273 [M+H]^+^	184.12118	C_9_H_16_N_2_O_2_	Cyclo(Ala-Ile)
10	5.05	209.1525 [M+Na]^+^	186.16198	C_11_H_22_O_2_	Undecanoic acid
11	5.17	353.1375 [M+Na]^+^	330.14672	C_19_H_22_O_5_	Imperanene
12	5.65	371.1480 [M+H]^+^	370.14164	C_21_H_22_O_6_	Fragransol D
13	5.78	401.1584 [M+H]^+^	400.15220	C_22_H_24_O_7_	Aschantin
14	6.19	265.1426 [M+H]^+^	264.13616	C_15_H_20_O_4_	1,10 : 4,5-diepoxy-germacren-12,8-olide
15	6.29	321.2415 [M+H]^+^	320.23514	C_20_H_32_O_3_	ent-16*α*,17-Hydroxy-19-kauranoic acid
16	6.49	323.2573 [M+H]^+^	322.25079	C_20_H_34_O_3_	Darutigenol
17	6.81	597.1969 [M+H]^+^	596.18938	C_31_H_32_O_12_	Candidate mass C_31_H_32_O_12_
18	7.00	211.0955 [M+H]^+^	210.08921	C_11_H_14_O_4_	Ethyl-5-ethoxy-2-hydroxy benzoate
19	7.30	443.1072 [M+H]^+^	442.09938	C_33_H_14_O_2_	Candidate mass C_33_H_14_O_2_
20	7.37	425.1582 [M+Na]^+^	402.16785	C_22_H_26_O_7_	Neociwujiaphenol
21	7.97	621.2307 [M+Na]^+^	598.24141	C_32_H_38_O_11_	Candidate mass C_32_H_38_O_11_
22	8.30	303.2311 [M+H]^+^	302.22458	C_20_H_30_O_2_	(E)-Labda-8(17),12-diene-15,16-dial
23	8.69	209.1161 [M+H]^+^	208.10994	C_12_H_16_O_3_	Elemicin
24	9.04	537.2112 [M+H]^+^	536.20463	C_30_H_32_O_9_	Isolappaol A
25	9.16	447.2015 [M+H]^+^	446.19407	C_24_H_30_O_8_	Lirioresinol B dimethyl ether
26	9.28	457.1844 [M+H]^+^	456.17842	C_25_H_28_O_8_	Kushenol G

## Data Availability

The raw data used to support the findings of this study are accessible from the corresponding author.
